# Fibronectin type III domain-containing protein 5/irisin in extracellular vesicles is reduced in older individuals

**DOI:** 10.1093/braincomms/fcag085

**Published:** 2026-04-03

**Authors:** Tai R Hunter, Natalia M Lyra E Silva, Guilherme B de Freitas, Tayna Rody, Luis E Santos, Jieming Zhang, Alexis Bullock, Maria Carmela Tartaglia, Sheela Abraham, Chiara Nicolini, Aimee J Nelson, Jennifer J Heisz, Margaret Fahnestock, Fernanda G De Felice

**Affiliations:** Department of Biomedical and Molecular Sciences, Queen’s University, Kingston, ON K7L 2V5, Canada; Department of Biomedical and Molecular Sciences, Queen’s University, Kingston, ON K7L 2V5, Canada; Centre for Neuroscience Studies, Queen’s University, Kingston, ON K7L 2V5, Canada; Centre for Neuroscience Studies, Queen’s University, Kingston, ON K7L 2V5, Canada; Institute of Medical Biochemistry Leopoldo de Meis, Federal University of Rio de Janeiro, Cidade Universitária, Rio de Janeiro, RJ 21941-902, Brazil; D’OR Institute for Research and Education, Rua Diniz Cordeiro, Rio de Janeiro, RJ 22281-100, Brazil; Department of Biomedical and Molecular Sciences, Queen’s University, Kingston, ON K7L 2V5, Canada; Department of Kinesiology, McMaster University, Hamilton, ON L8S 4K1, Canada; Tanz Centre for Research in Neurodegenerative Diseases, University of Toronto, Toronto, ON M5S 1A8, Canada; Department of Biomedical and Molecular Sciences, Queen’s University, Kingston, ON K7L 2V5, Canada; Department of Kinesiology, McMaster University, Hamilton, ON L8S 4K1, Canada; Department of Kinesiology, McMaster University, Hamilton, ON L8S 4K1, Canada; Department of Kinesiology, McMaster University, Hamilton, ON L8S 4K1, Canada; Department of Psychiatry and Behavioural Neurosciences, McMaster University, Hamilton, ON L8N 3K7, Canada; Department of Biomedical and Molecular Sciences, Queen’s University, Kingston, ON K7L 2V5, Canada; Centre for Neuroscience Studies, Queen’s University, Kingston, ON K7L 2V5, Canada; Institute of Medical Biochemistry Leopoldo de Meis, Federal University of Rio de Janeiro, Cidade Universitária, Rio de Janeiro, RJ 21941-902, Brazil; D’OR Institute for Research and Education, Rua Diniz Cordeiro, Rio de Janeiro, RJ 22281-100, Brazil

**Keywords:** Alzheimer’s disease, biomarkers, exerkines, exercise, ageing

## Abstract

The progressive accumulation of physiological stress as we age, known as allostatic load, is linked to an increased risk of dementia. Fostering brain resilience through physical exercise can counteract allostatic load and improve adaptation to age-related brain alterations. Fibronectin type III domain-containing protein 5 (FNDC5)/irisin is a neuroprotective exercise-linked hormone found in extracellular vesicles (EV-FNDC5/irisin). Here, we sought to analyse EV-FNDC5/irisin in ageing as a promising biomarker of brain resilience.

We measured exercise-associated factors, including EV-FNDC5/irisin, brain-derived neurotrophic factor (BDNF), and cathepsin B in the serum of 31 young (18–28 years) and 19 older subjects (65–79 years).

Levels of FNDC5/irisin in serum-derived EVs are markedly reduced in older subjects compared to young (*P* = 0.004). We report a reduction in nanoparticles isolated from the serum of older participants (*P* = 0.009). While EV-FNDC5/irisin positively correlates with BDNF in young subjects (Spearman r = 0.40, *P* = 0.038), this correlation is absent in elderly subjects (Spearman r = −0.25, *P* = 0.34).

This study provides initial evidence that EV-FNDC5/irisin is reduced in older individuals and loses correlation with BDNF. Our identification of peripheral, exercise-linked factors associated with age may inform biomarker discovery and interventions to promote brain resilience.

## Teaser

Irisin, an exercise-induced hormone with neuroprotective effects, was recently identified in extracellular vesicles (EVs). Hunter et al. show that irisin is reduced in the peripheral EVs of older subjects compared to young, suggesting that EV-irisin may be a novel blood biomarker for ageing and brain resilience.

## Graphical Abstract

**Graphical Abstract:**
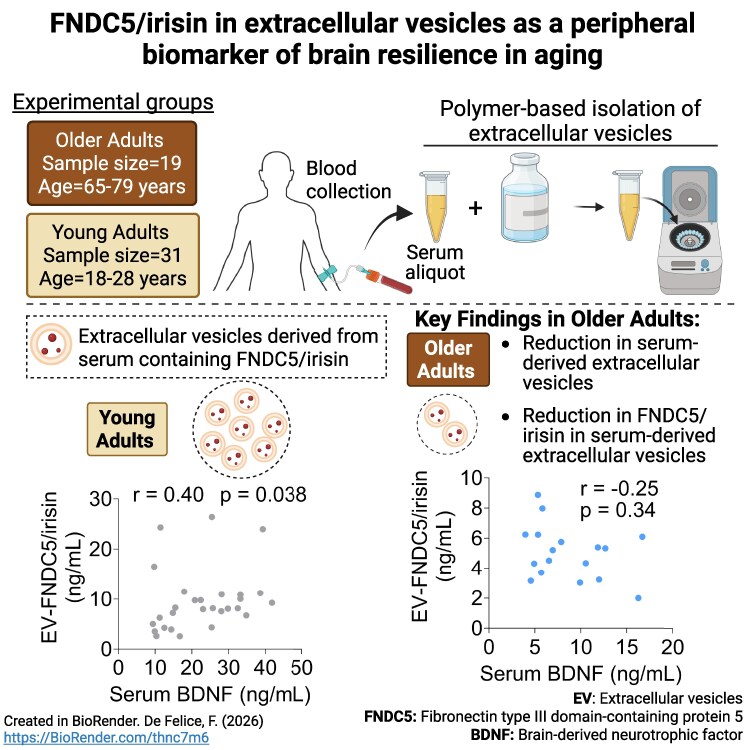

## Introduction

As we age, we are subject to cumulative physiological stress due to repeated exposure to physical, emotional, and environmental stressors. The chronic, progressive impact, known as allostatic load, contributes to age-related declines, increasing the risk of age-related and chronic conditions.^1^ In the brain, allostatic overload can manifest as chronic inflammation, oxidative stress, and structural and functional abnormalities, all of which increase the risk of Alzheimer’s disease.^2^ A key strategy to mitigate the impact of allostatic load throughout the lifespan is to improve brain resilience, which enhances the capacity to resist unhealthy brain ageing.^3^ Older adults with greater brain resilience are expected to be less vulnerable to age-related brain changes. Social and lifestyle interventions can strengthen resilience, and identifying biomarkers of brain resilience may offer new strategies to reduce the burden of Alzheimer’s disease and dementia.

Physical exercise is known to promote healthy brain ageing and to both delay the onset of and alleviate dementia associated with Alzheimer’s disease.^4,5^ The beneficial effects of exercise on cognition are mediated in part by signalling molecules, called myokines, released by skeletal muscle cells following exercise.^6^ Irisin, which is cleaved from fibronectin type III domain-containing protein 5 (FNDC5), is a myokine initially described for mediating the beneficial effects of exercise on adipocyte metabolism.^7^ Interestingly, FNDC5/irisin is reduced in the hippocampi and cerebrospinal fluid (CSF) of Alzheimer’s disease patients compared to age-matched cognitively unimpaired controls.^8^ In Alzheimer’s disease mouse models, FNDC5/irisin upregulates hippocampal brain-derived neurotrophic factor (BDNF) and rescues synaptic plasticity and memory deficits, which is the mechanism thought to underlie its neuroprotective effects.^9-11^

In recent years, evidence for a new mechanism underlying the physiological benefits of physical exercise has emerged: the stimulated secretion of extracellular vesicles (EVs).^12^ In addition to their role in mediating routine intra- and intercellular communication, EVs released into circulation upon exercise carry myokines and are proposed to mediate crosstalk between the periphery and the brain.^6,13^ It has recently been shown that chronic exercise upregulates EV-FNDC5/irisin in humans and mice.^14,15^ Our group further showed that a chronic exercise protocol increases FNDC5/irisin in the brains of mice.^10^

Altered levels of myokines and their downstream targets are associated with ageing and Alzheimer’s disease. FNDC5/irisin is reduced in the CSF and hippocampi of Alzheimer’s disease and in the circulation of older adults.^10,16,17^ Similarly, circulating levels of BDNF also decline with increasing age and in Alzheimer’s disease.^18-20^ Our group previously reported a positive correlation between CSF irisin with BDNF, amyloid-beta (Aβ)42 and cognition in Alzheimer’s disease, suggesting that CSF irisin may be a useful biomarker in Alzheimer’s disease.^8^ Whether circulating EV-FNDC5/irisin also changes with age or in Alzheimer’s disease remains to be determined.

Another EV-associated myokine implicated in Alzheimer’s disease is cathepsin B, a lysosomal cysteine protease.^21^ In a study by Moon *et al*., cathepsin B enhanced hippocampal BDNF expression *in vitro* and exercise-induced cathepsin B correlated with memory function in young humans.^22^ However, its role in Alzheimer’s disease pathogenesis is complex. In the familial form of the disease, cathepsin B has anti-amyloidogenic effects via the proteolytic cleavage of Aβ peptides.^23-25^ On the other hand, in sporadic Alzheimer’s disease, several studies suggest that cathepsin B can cleave amyloid precursor protein (APP) to form Aβ, and that cathepsin B inhibition is therapeutic.^26,27^ Higher levels of circulating cathepsin B are also associated with age and Alzheimer’s disease.^28-30^ Interestingly, cathepsin B in plasma EVs correlates with amyloid positivity in middle-age and older adults.^21^ Hence, the association of circulating cathepsin B with ageing and Alzheimer’s disease remains unclear.

Recent technological developments have allowed the detection of Alzheimer’s disease biomarkers in peripheral blood.^31^ Sampling and analysing blood addresses limitations associated with the traditional neuroimaging and CSF biomarkers, such as high costs, invasiveness and low accessibility.^31^ The measurement of Aβ species in the blood can be used to identify brain Aβ deposition in Alzheimer’s disease.^32^ Furthermore, biomarkers such as glial fibrillary acidic protein (GFAP),^33,34^ neurofilament light (NfL)^35^ and total tau (t-tau)^36^ in the blood correlate with neurodegeneration and age-related cognitive decline. Recent studies indicate that the measurement of cargo packed within neuronal-derived EVs may also be a future avenue to detect core and non-core Alzheimer’s disease biomarkers in the periphery.^37-39^

EVs can also communicate cellular senescence; senescent cells, which have exited the cell cycle, release EVs containing protein and microRNAs that can induce senescence in healthy neighbouring cells.^40,41^ Senescence-associated EVs can contribute to the development of age-related diseases such as osteoporosis through the inhibition of osteogenesis^42^ and Alzheimer’s disease through the propagation of neurotoxic Aβ and tau proteins.^43,44^ However, the exact role of EVs in ageing remains to be determined.

Based on the recent evidence that EVs carry myokines, including FNDC5/irisin, and that EVs mediate intra- and inter-tissue communication in healthy ageing as well as in age-related diseases, we aimed to investigate the levels of EV-FNDC5/irisin in young and older subjects. Given the potential of FNDC5/irisin as a biomarker of brain resilience, we investigated correlations between EV-FNDC5/irisin and serum BDNF in young and older subjects.

## Materials and methods

### Study approval and ethics

All participants received an honorarium for their participation and provided written informed consent prior to their inclusion in the study. All experimental protocols were in accordance with the Declaration of Helsinki and approved by the Hamilton Integrated Research Ethics Board (13–508).

### Human subjects

Thirty-one healthy young participants (14 men and 17 women; age range = 18–28 years, mean = 21.6) were recruited through posters distributed to McMaster University students on campus and online. The exclusion criteria for young subjects included smoking, the presence of medical conditions, a known history of neurological disease, the use of street drugs or medications, structured exercise >1 h/week and, for females, the use of hormonal contraceptives/supplements. Nineteen older subjects (9 men and 10 women; age range = 65–79 years, mean = 70.7) were recruited through posters and local news outlets in Hamilton and surrounding areas. Older participants met the inclusion criteria if they were 65 years of age or older; free from cognitive impairment, auto-immune disease, type II diabetes mellitus and obesity (BMI > 35); non-smokers; and not currently on hormone replacement therapy or taking beta-blocker medications. Older participants reported completing an average of 4.4 h/week of any physical activity. The Montreal Cognitive Assessment (MoCA) test, used to assess cognition in the older group, was administered by a trained researcher. As all participants had >12 years of education, the MoCA education correction was never applied during scoring.

### Blood collection and serum processing

Peripheral blood samples were collected between 8:00 and 11:00 AM following 12 h of fasting. Older participants were instructed to avoid engaging in vigorous exercise and avoid consumption of alcohol and caffeine for 24 h prior to their scheduled visit. Serum samples were collected into BD Vacutainer Serum (young) or SST (older) tubes (BD, Franklin Lanes, NJ). Young participant samples were left to clot at room temperature for 45 min and subsequently centrifuged at 3488 × *g* for 10 min at 4°C. Older participant samples were allowed 30 min to clot at room temperature and then centrifuged at 1000 × *g* for 15 min at 4°C. Since the young and older participants were recruited from separate study cohorts, the sample processing varied slightly. The supernatants were aliquoted into 1.5 mL Eppendorf tubes and stored at −80°C (young participant samples) or −20°C (older participant samples) until analysis.

### EV isolation

Frozen serum samples were thawed at 4°C. ExoQuick-ULTRA EV Isolation System (EQULTRA-20A-1, System Biosciences, Palo Alto, CA, USA) was used to isolate and purify EVs from 250 uL of serum according to the manufacturer’s instructions. EVs were aliquoted and stored at −80°C.

### ELISA

Serum and EV samples were thawed at 4°C. EVs were pre-treated with RIPA Lysis and Extraction Buffer (Thermo Fisher Scientific, Waltham, MA, USA) to enhance the detection of EV-cargo. Human FNDC5/Irisin DuoSet enzyme linked immunosorbent assay (ELISA) (DY008, R&D Systems, Minneapolis, MN, USA), Human Cathepsin B ELISA (ab272205, Abcam, Cambridge, UK), and Human/Mouse BDNF DuoSet ELISA (DY248, R&D Systems) kits were used according to the manufacturer’s instructions. All standards and samples were analysed in duplicate. The optical density was determined at 450 nm with wavelength correction set at 540 nm. The SpectraMax ABS Plus plate reader (Molecular Devices, San Jose, CA, USA) was used for FNDC5/irisin and cathepsin B quantification, and the Tecan Infinite M1000 PRO plate reader (Tecan Group Ltd., Männedorf, Switzerland) was used for BDNF quantification. In measurements made in EV samples, concentrations were normalized to the volume of blood used to isolate EVs.

### Nanoparticle tracking analysis

Nanoparticle tracking analysis (NTA) was performed using the ZetaView® PMX 110 V3.0 (Particle Metrix GmbH, Meerbusch, Germany) to assess the size and concentration of nanoparticles isolated from serum. The auto-alignment of the instrument was performed using 100 nm polystyrene beads. EVs were thawed once at 4°C, diluted between 250× and 5000× in PBS, and injected into the instrument. Readings were performed in triplicate using the following settings: sensitivity 80, temperature 23°C, shutter 100, positions 11, and filter wavelength scatter. For quality control, at least 500 particles and 8 accepted positions were a minimum requirement for each experiment. Particles >500 nm were excluded from the calculation of EV particle concentration.

### Single-molecule array

Serum biomarkers were measured by single-molecule array (SIMOA) on an SR-X instrument (Quanterix, Lexington, MA, USA). Quanterix Neurology 3-Plex A, p-Tau181 V2 and Neurology 2-Plex B kits were used to measure levels of NfL, GFAP, p-tau181 and t-tau, following the manufacturer’s instructions. Two bridge samples were included on every plate and used to assess comparability between runs. Average inter-plate CVs were 8.6% for NfL, 11.3% for GFAP, 15.9% for p-tau181 and 5.8% for t-tau.

### Statistics

Statistical analyses were performed using GraphPad Prism 10.2.3 (GraphPad Software Inc., La Jolla, CA, USA). Variables were checked for normal distribution using the D’Agostino and Pearson normality test. Groups were compared using two-tailed unpaired Student’s *t*-test or Fisher’s exact test (*α* = 0.05). Data are expressed as the mean ± standard deviation (SD). Only subjects with corresponding complete data were included for correlation analyses. Correlations were established using the Spearman correlation coefficient.

## Results

### Sample characteristics

Demographic characteristics of the 50 included subjects are presented in **Table 1**. The average ages of the young and older groups were 21.6 ± 2.8 and 70.7 ± 4.6 years, respectively. MoCA results indicate that the older group consisted of a mix of participants, those who scored ≥26 out of 30, suggesting normal cognition (*n* = 11), and those who scored 18–25 out of 30, suggesting possible mild cognitive impairment (*n* = 8). Serum levels of Aβ42, Aβ40, t-tau, p-tau181, NfL, and GFAP for both cohorts are reported in **Table 2**. In the older group, we observed no correlation between MoCA scores and Alzheimer’s disease-related biomarker levels (Supplementary Data 1).

**Table 1:** Demographic characteristics of subjects

	Young group (*n* = 31)	Older group (*n* = 19)	*P*-value
Sex, male/female	14/17	9/10	>0.9999
Age (years)	21.6 ± 2.8 (18–28)	70.7 ± 4.6 (65–79)	<0.0001
MoCA	N/A	26 ± 2.5 (19–29)	
Education (years)	N/A	18 ± 3.5 (12–25)	
Weight (kg)	N/A	74.9 ± 11.1 (57.8–90.6)	
BMI	N/A	26.1 ± 2.51 (20.4–31.6)	

Values are presented as means ± SD (min–max). *P*-values calculated from two-tailed unpaired Student’s *t*-test, except for sex, which was analysed using Fisher's exact test.

Abbreviations: MoCA, Montreal Cognitive Assessment; BMI, body mass index.

**Table 2:** Alzheimer's disease-associated biomarker and myokine characteristics of subjects

	Young group (*n* = 31)	Older group (*n* = 19)	*P*-value
Serum Aβ42 (pg/mL)	N/A	9.589 ± 2.307 (5.205–12.60)	
Serum Aβ40 (pg/mL)	N/A	281.8 ± 130.2 (87.22–667.7)	
Serum Aβ42/Aβ40	N/A	0.03801 ± 0.01157 (0.01807–0.05968)	
Serum t-tau (pg/mL)	0.5135 ± 0.2552 (0.1579–1.137)	0.8368 ± 0.4706 (0.3780–2.157)	0.0127
Serum p-tau181 (pg/mL)	1.024 ± 1.095 (0–4.833)	1.040 ± 0.657 (0.1217–2.353)	0.9599
Serum NfL (pg/mL)	3.975 ± 1.476 (1.540–7.072)	15.82 ± 4.828 (9.906–26.89)	<0.0001
Serum GFAP (pg/mL)	52.18 ± 22.55 (20.11–110.6)	188.6 ± 103.4 (66.50–442.7)	<0.0001
Particles/mL	1.698 × 10^11^ ± 1.0515 × 10^11^ (1.780 × 10^10^–3.790 × 10^11^)	9.033 × 10^10^ ± 9.211 × 10^10^ (1.460 × 10^10^–3.970 × 10^11^)	0.0092
EV-FNDC5/irisin (ng/mL)	9.626 ± 6.226 (2.563–26.40)	5.015 ± 1.738 (2.001–8.878)	0.0038
EV-cathepsin B (ng/mL)	0.3759 ± 0.6152 (0–1.662)	1.603 ± 0.4120 (1.223–2.770)	<0.0001
Serum cathepsin B (ng/mL)	50.41 ± 15.55 (28.28–99.49)	102.8 ± 44.52 (48.01–197.0)	<0.0001
Serum BDNF (ng/mL)	24.48 ± 13.88 (9.382–76.80)	8.686 ± 3.951 (3.964–16.70)	<0.0001

Values are presented as means ± SD (min – max). *P*-values calculated from two-tailed unpaired Student’s *t*-test.

Abbreviations: Aβ40, amyloid-β40; Aβ42, amyloid-β42; t-tau, total tau; NfL, neurofilament light; GFAP, glial fibrillary acidic protein; EV, extracellular vesicle; BDNF, brain-derived neurotrophic factor.

### EV-FNDC5/irisin is reduced in older subjects

**Figure 1:**
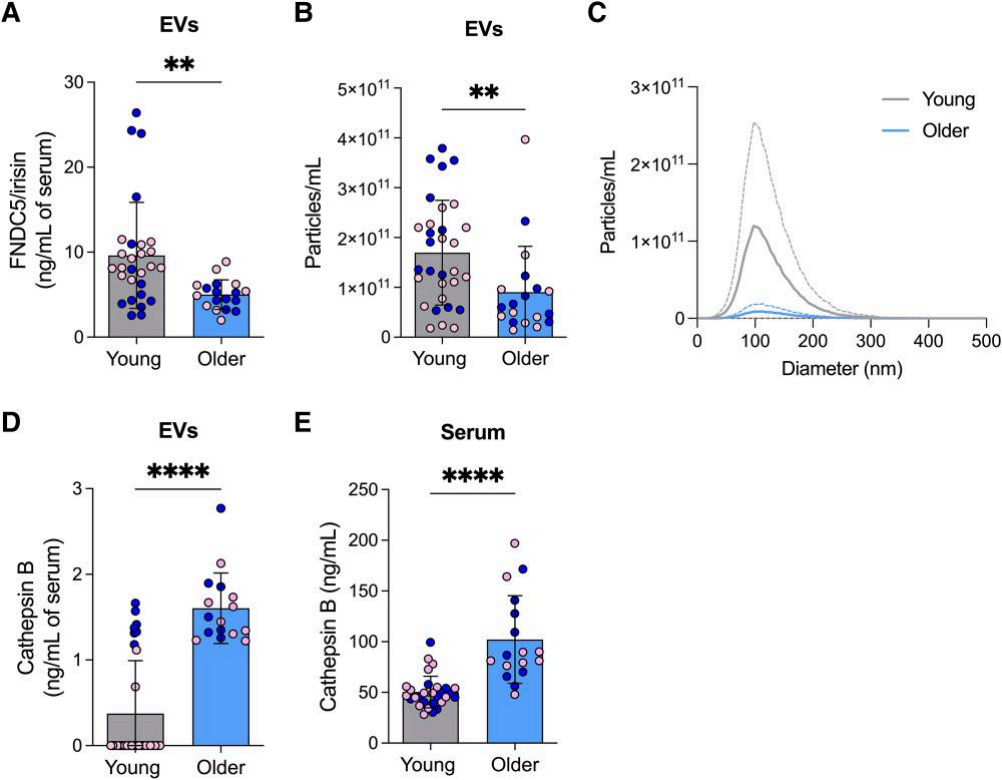
**EV-FNDC5/irisin is reduced in older subjects.** (**A**) ELISA quantification of FNDC5/irisin levels in serum EVs of young (*n* = 28) and older subjects (*n* = 18). Females and males represented by pink and dark blue symbols, respectively. Bars represent mean ± SD. (**B**) Nanoparticle concentration of EVs isolated from young (*n* = 31) and older subjects (*n* = 19) measured by NTA. Females and males represented by pink and dark blue symbols, respectively. Bars represent mean ± SD. (**C**) Nanoparticle concentration by diameter of EVs isolated from young (grey; *n* = 31) and older (blue; *n* = 19) subjects determined by NTA. Solid lines represent the mean and dotted lines represent ± SD. (**D**) ELISA quantification of cathepsin B levels in serum-derived EVs of young (*n* = 31) and older subjects (*n* = 16). Females and males represented by pink and dark blue symbols, respectively. Bars represent mean ± SD. (**E**) ELISA quantification of cathepsin B in serum of young (*n* = 30) and older subjects (*n* = 17). Females and males represented by pink and dark blue symbols, respectively. Bars represent mean ± SD. EVs = extracellular vesicles. The result of a two-tailed unpaired *t*-test (*α* = 0.05) is indicated above the plots. **P ≤ 0.0; ****P < 0.0001.

In light of previous findings that EV-FNDC5/irisin exerts protective effects in vascular ageing and adipocyte metabolism,^14,15^ we initially asked whether EV-FNDC5/irisin is changed in ageing. EVs were isolated from serum and characterized by nanoparticle count and myokine levels **(Table 2)**. Interestingly, older participants had significantly reduced FNDC5/irisin levels in serum EVs compared to the young cohort (*P* = 0.004, two-tailed unpaired *t*-test; *Hedges’ g* = 0.91; Fig. 1A) as well as fewer serum-isolated nanoparticles (*P* = 0.009, two-tailed unpaired *t*-test; *Hedge’s g* = 0.78; Fig. 1B and C).

We also evaluated the levels of another EV-associated myokine, cathepsin B. Levels of EV-cathepsin B were elevated in older subjects (*P* < 0.0001, two-tailed unpaired *t*-test; *Hedge’s g* = 2.17; Fig. 1D), indicating that the modulation of myokines levels in EVs is not solely driven by the reduction in nanoparticle concentration associated with age. Furthermore, we observed an increase in cathepsin B in the serum of older subjects (*P* < 0.0001, two-tailed unpaired *t*-test; *Hedge’s g* = 1.77; Fig. 1E).

### EV-FNDC5/irisin correlates with BDNF in young, but not in older subjects

**Figure 2:**
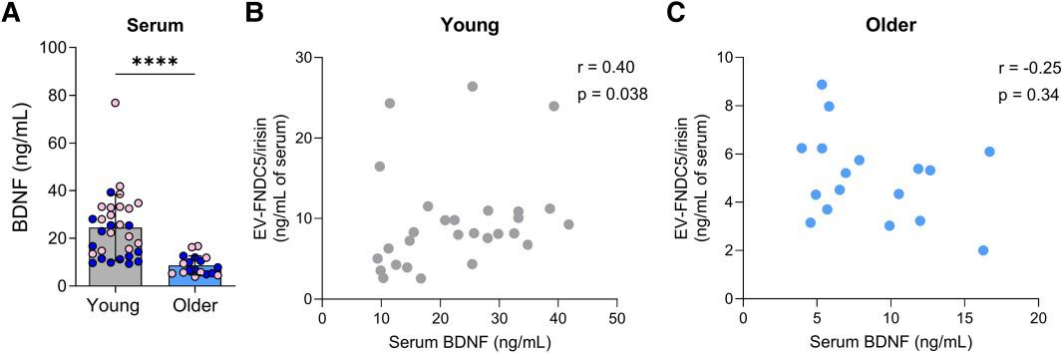
**Reduction in serum BDNF in older subjects is not correlated with EV-FNDC5/irisin.** (**A**) ELISA quantification of BDNF in serum of young (*n* = 31) and older subjects (*n* = 18). Females and males represented by pink and dark blue symbols, respectively. Bars represent mean ± SD. The result of a two-tailed unpaired *t*-test (*α* = 0.05) is indicated above the plot. (**B, C**) Correlation between EV-FNDC5/irisin and serum BDNF in young (*n* = 28) (**B**) and older (*n* = 17) (**C**) subjects. The results of Spearman’s correlation are indicated in the plots. Each data point represents an individual subject. EV = extracellular vesicle. ****P < 0.0001.

Since FNDC5/irisin induces BDNF, we next investigated this correlation in human subjects. BDNF was significantly reduced in the serum of older participants (*P* < 0.0001, two-tailed unpaired *t*-test; *Hedge’s g* = 1.37; Fig. 2A). Serum BDNF showed a significant positive correlation with EV-FNDC5/irisin in young subjects (Spearman *r* = 0.40, *P* = 0.038; Fig. 2B). Interestingly, this correlation was absent in older subjects (Spearman *r* = -0.25, *P* = 0.34; Fig. 2C). Altogether, these findings suggest that reductions in EV-FNDC5/irisin in older age may also be accompanied with dysregulations in FNDC5/irisin-BDNF signalling.

## Discussion

Recent evidence suggests that exercise enriches FNDC5/irisin in circulating EVs, which mediates the beneficial effects of exercise on vascular ageing and adipocyte metabolism.^14,15^ Understanding how EV-FNDC5/irisin, along with other exercise-linked factors, varies with age is beneficial for identifying therapeutic and biomarker targets for age-related neurodegeneration. To the best of our knowledge, this is the first report showing a reduction in circulating EV-FNDC5/irisin in ageing.

Previous studies on age-related changes in EV concentration show contrasting results. A cross-sectional study using density cushion centrifugation followed by size-exclusion chromatography to isolate EVs from plasma found no change in EV concentration with age,^45^ while a longitudinal study using a polymer-based method (ExoQuick-ULTRA) reported a decline in plasma EVs with age.^46^ Using ExoQuick-ULTRA to isolate EVs from serum, we found that older subjects had significantly fewer EVs compared to the young. While precipitation is a simple, high-yield method, it results in low purity, unlike ultracentrifugation and size-exclusion chromatography, which are very pure but require large volumes of starting material and have low recovery.^47^ Furthermore, differences in serum and plasma preparation (i.e. anticoagulants and clotting) affect the concentration of blood-derived EVs.^48^ In addition to the methods used, an increase in EV internalization by aged cells may partially explain the lower levels of circulating EVs observed in older individuals.^46^ The association between circulating EV levels and cognition, as well as age-associated differences in the EV-FNDC5/irisin response to exercise, must be clarified by future studies with larger sample sizes.

Exercise can ameliorate age-related cognitive decline through the PGC-1α-FNDC5-BDNF signalling pathway.^9^ In the CSF, levels of irisin positively correlate with levels of BDNF.^8^ Since EVs may act as transporters for circulating FNDC5/irisin, promoting the beneficial effects of exercise on memory, it is important to establish the relationships between EV-FNDC5/irisin and BDNF in the periphery. Here, we report a decline in circulating BDNF in older people, in agreement with previous reports.^18,20^ We further demonstrate a direct correlation between circulating BDNF and EV-FNDC5/irisin in young subjects.^9^ The loss of the correlation between BDNF and EV-FNDC5/irisin in older subjects suggests an age-related dysregulation of this pathway or a diminished capacity of FNDC5/irisin-containing EVs to reach the brain. It is important to note that several factors such as sex, exercise and age can impact plasma BDNF levels.^49^ Accordingly, our findings should be interpreted as preliminary, and future mechanistic studies and larger cohorts will be required to further investigate the relationship between EV-FNDC/irisin and BDNF.

Higher levels of circulating cathepsin B are associated with age and Alzheimer’s disease.^28,29,50^ In a study by Yuyama and colleagues, EV-associated cathepsin B was found to correlate with Alzheimer’s disease-related biomarker progression, suggesting that it may be a promising biomarker or therapeutic target in Alzheimer’s disease.^21^ Consistent with this study, we detected cathepsin B in the serum EVs of older adults. However, we were unable to consistently detect cathepsin B in EVs of young adults, likely due to its low concentration in total serum. More sensitive detection methods are needed to determine the association between EV-associated cathepsin B, ageing and Alzheimer’s disease biomarkers. Additionally, we report that serum cathepsin B levels are elevated in older adults, in alignment with previous studies.^30,51^ While cathepsin B has been identified as a myokine linked to memory function in young individuals, its role in ageing may differ.^22^

A limitation of this study is the modest sample size, which was constrained by the availability of serum samples from a previously established cohort. As a result, statistical power is limited. Additionally, since our older group consists of a mixed population, we cannot fully exclude the possibility that underlying pathology associated with possible mild cognitive impairment in the older group contributes to the differences observed between the young and older groups.

In conclusion, these findings point towards EV-FNDC5/irisin as a novel blood biomarker candidate for brain resilience and age-related risk for neurodegeneration. The identification of age- and Alzheimer’s disease-associated myokines can help elucidate the role of physical activity in preventing age-related neurodegeneration and guide prevention strategies in ageing adults or those with pre-clinical Alzheimer’s disease.

## Supplementary Material

fcag085_Supplementary_Data

## Data Availability

The authors confirm that the data supporting the findings of this study are available within the article and Supplementary Data 2.
